# Direct-to-Implant Prepectoral Breast Reconstruction with a Novel Collagen Matrix Following Nipple-Sparing Mastectomy: A Case Report

**DOI:** 10.3390/reports8030120

**Published:** 2025-07-24

**Authors:** Josip Banović, Zrinka Pribudić, Mia Buljubašić Madir, Vedran Beara, Luka Perić, Marija Čandrlić, Željka Perić Kačarević

**Affiliations:** 1 Department of Plastic and Reconstructive Surgery, Split University Hospital Center, Spinčićeva ulica 1, 21000 Split, Croatiavedran.beara@kbsplit.hr (V.B.); 2 Department of Nuclear Medicine and Oncology, Faculty of Medicine Osijek, J.J. Strossmayer University of Osijek, 31000 Osijek, Croatia; 3 Department of Integrative Dental Medicine, Faculty of Dental Medicine and Health Osijek, J.J. Strossmayer University of Osijek, 31000 Osijek, Croatia; 4 Department of Anatomy, Histology, Embriology, Pathology Anatomy and Pathology Histology, Faculty of Dental Medicine and Health Osijek, J.J. Strossmayer University of Osijek, 31000 Osijek, Croatia; 5 The botiss biomaterials GmbH, Ullsteinstrasse 108, 12109 Berlin, Germany

**Keywords:** breast cancer, case report, collagen matrix, reconstruction

## Abstract

**Background and Clinical Significance**: Breast reconstruction following mastectomy is a critical aspect of treatment for many patients, offering both physical and psychological benefits. Traditional methods include autologous tissue flaps and implants, with implant-based techniques being the most prevalent in the Western world. However, complications such as capsular contracture remain a concern. Acellular dermal matrices (ADM) have emerged as a valuable alternative, improving outcomes by reducing capsular contracture rates and enhancing tissue integration. **Case Presentation**: This case report presents the first use of a novel ADM, biocade^®^ (biotrics bioimplants AG, Berlin, Germany) in breast reconstruction following a mastectomy. A 55-year-old female patient underwent a left-sided nipple-sparing mastectomy, followed by prepectoral direct-to-implant reconstruction using an ADM-wrapped implant. The patient tolerated the procedure well, with no immediate complications observed. Postoperative monitoring focused on wound healing and assessing for signs of complications related to the implant. The use of the ADM resulted into satisfactory aesthetic and functional outcomes. **Conclusions**: The successful outcome of this case highlights the potential benefits of using collagen matrices in breast reconstruction, particularly in preserving mastectomy scenarios. The immediate results and improved aesthetics offered by prepectoral direct-to-implant reconstruction with ADM align well with patient expectations for a more natural appearance and faster recovery. However, this case report also highlights the need for ongoing research to fully explore the potential of these biomaterials and address associated challenges.

## 1. Introduction and Clinical Significance

Breast cancer is the most common malignancy among women, with approximately one in eight women developing invasive breast cancer over their lifetime. As of 2020, it remains the most frequently diagnosed cancer globally [1]. Traditional breast cancer treatment has evolved from radical mastectomies to more conservative approaches like skin-sparing and nipple-sparing procedures, depending on the extent of cancer spread. In that context, surgical intervention may involve breast-conserving procedures, such as lumpectomy or quadrantectomy, which aim to remove the tumor with a margin of healthy tissue [2].

Breast implants are widely used in both aesthetic and reconstructive surgery. Recent population data from Italy estimate that approximately 4.1% of women aged 20–70 have breast implants, with 52.9% of these placed for reconstructive purposes, highlighting the clinical relevance of implant-based reconstruction [3]. Despite the growing number of procedures, breast implant surgery maintains a high safety profile, with large-scale epidemiological studies reporting a perioperative mortality rate of 0%, even when accounting for oncologic and non-oncologic indications [4]. These findings support the ongoing refinement of implant-based techniques, particularly direct-to-implant reconstruction, which offers both clinical and aesthetic benefits when combined with modern soft tissue support systems such as acellular dermal matrices (ADMs).

ADMs have played a significant role in improving the safety and efficacy of implant-based reconstruction [5]. Recently, a novel ADM named biocade^®^ (biotrics bioimplants AG, Berlin, Germany) has been introduced, designed to support surgeons in various reconstructive procedures. Key properties include rapid healing facilitated by early cell migration and revascularization, a short rehydration time, and excellent mechanical strength, being both tear-resistant and flexible [6,7]. In vivo studies have demonstrated successful vascularization and early integration due to its open-pore structure, which supports the formation of new tissue [8]. Considering these advancements, we aimed to address the gap in the literature with regard to the use of ADM in breast reconstruction, particularly in the context of sparing mastectomies. Therefore, in this case report, we present the first use of a biocade^®^ in breast reconstruction following a left-sided nipple-sparing mastectomy and its potential in such clinical scenario.

## 2. Case Presentation

### 2.1. Clinical Findings and Treatment Plan

This case report was not part of a clinical trial and did not involve experimental treatment. In accordance with Croatian legislation, specifically the Act on the Medical Profession (Official Gazette 121/03) and the Act on the Protection of Patients’ Rights (Official Gazette 169/04), this type of retrospective case report does not require prior approval by an ethics committee. Written informed consent was obtained from the patient for both the surgical treatment and the publication of anonymized clinical data and images. All ethical principles outlined in the Declaration of Helsinki, as well as the ICMJE and CARE guidelines, were followed.

A 55-year-old female patient presented at the Department of Plastic and Reconstructive Surgery, Clinical Hospital Split, with a palpable breast mass. The patient was otherwise healthy, with no known comorbidities, ongoing medications, or reported allergies. The diagnosis of breast cancer was made incidentally during a routine screening mammography performed as part of a national preventive program under the auspices of the Croatian Ministry of Health. The initial radiological suspicion of a tumor was subsequently confirmed by breast ultrasound, after which, a core needle biopsy was performed, providing histopathological verification of malignancy. Additional systemic staging, comprising chest X-ray, as well as radiographs of the spine, pelvis, and femurs, revealed no signs of distant metastasis. Finally, based on histopathological findings the tumor was characterized as estrogen receptor-positive and HER2-negative, with no lymph node involvement. Tumor staging was determined to be T1N0M0, indicating that the malignancy was confined to the breast, with no clinical or radiological evidence of regional lymph node involvement or distant metastasis.

Following diagnosis, a multidisciplinary breast cancer team convened and recommended surgical intervention as the first step in the treatment plan. Given the patient’s preference for breast conservation and the absence of skin or chest wall invasion, a left-sided nipple-sparing mastectomy was performed. To optimize aesthetic outcomes and minimize complications, a breast reconstruction was undertaken using an implant wrapped with a collagen matrix (biocade^®^). The decision to use biocade^®^ in this case was based on several factors, including its pliability, structural integrity, and absence of chemical cross-linkers or preservatives, which may support more favorable integration and reduce local inflammatory responses. Its non-preshaped design allowed for flexible adaptation around the implant, and the product’s terminal sterilization and freeze-dried preservation contributed to ease of intraoperative handling. The choice was also influenced by product availability and the surgical team’s interest in assessing its clinical applicability in prepectoral direct-to-implant breast reconstruction. This collagen matrix, analogous to human dermis, is derived from native porcine collagen and it is a CE-marked Class III medical device approved in Europe since 2021, in accordance with Directive 93/42/EEC. Furthermore, biocade^®^ has a thickness ranging from 1.2 to 1.7 mm (dry to hydrated state). It is preserved by freeze-drying without the use of preservatives or cross-linking agents, requiring rehydration before clinical use. The device has a shelf life of 3 years, and biocade^®^ is designed to be fully resorbed within approximately three months following implantation. This estimate is supported by preclinical data demonstrating progressive membrane degradation and integration over time [9], although, it is well known that resorption kinetics may vary depending on anatomical placement and individual host response [10,11]. It should be stored in its original packaging in a dry environment at temperatures between +5 °C and +30 °C. The expiration date is printed on both the box and the sterile inner packaging. It offers high biomechanical stability, lot-to-lot uniformity, and terminal sterilization, reducing the risk of infection [9].

The patient underwent detailed preoperative counseling, during which the surgical procedure, expected outcomes, and potential complications were thoroughly discussed. The benefits of nipple-sparing mastectomy with prepectoral direct-to-implant reconstruction using a collagen matrix were explained. The patient was also informed about potential risks, including infection, implant-related complications, and aesthetic concerns. This detailed discussion ensured that the patient provided informed consent for the procedure.

### 2.2. Surgical Procedure

#### 2.2.1. Preoperative Assessment

**Figure 1:**
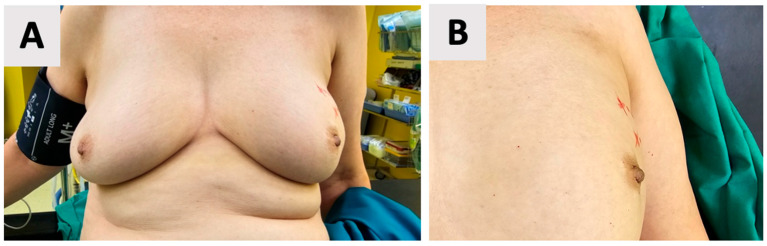
Preoperative skin markings for a left-sided nipple-sparing mastectomy. (**A**) Overview of the left breast with red line markings outlining the incision site and areas to be preserved, including the nipple-areolar complex. (**B**) Close-up view of the markings, demonstrating precise delineation of the surgical boundaries.

Accurate anthropometric measurements of the left breast were obtained, including the craniocaudal and laterolateral base width as well as the nipple projection. These measurements also guided the surgical approach, namely, the selection of optimal implant dimensions and the precise positioning of incisions for a tailored fit and predictable aesthetic outcome. Accordingly, skin markings were made to delineate the incision site and identify areas to be preserved, including the nipple-areolar complex. These markings served as a guide for the surgical approach, ensuring that the aesthetic and oncological goals were met (Figure 1).

#### 2.2.2. Mastectomy

**Figure 2:**
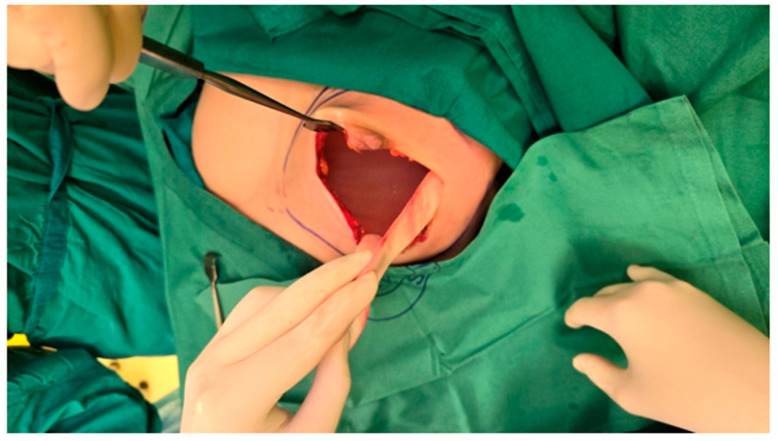
Intraoperative view following left-sided nipple-sparing mastectomy and assessment of implant size and symmetry before final implant placement. The figure shows dilated skin envelope with a trial breast implant in place and the preserved nipple-areolar complex.

The mastectomy was conducted with careful attention to maintaining the integrity of the preserved skin and nipple-areolar complex, which is crucial for optimal aesthetic outcomes. Therefore, a left-sided nipple-sparing mastectomy was performed, involving the removal of all breast tissue while preserving the skin and nipple-areolar complex. Then the pocket of the implant was created for adequate space during reconstruction. Regarding the selection of the implant, the appropriate size was determined based on the patient’s anatomical measurements, desired breast volume, and the space created during the mastectomy. Trial implants were used to confirm the desired size and symmetry before proceeding with the final implant placement (Figure 2).

#### 2.2.3. Implant Placement and Wound Closure

**Figure 3:**
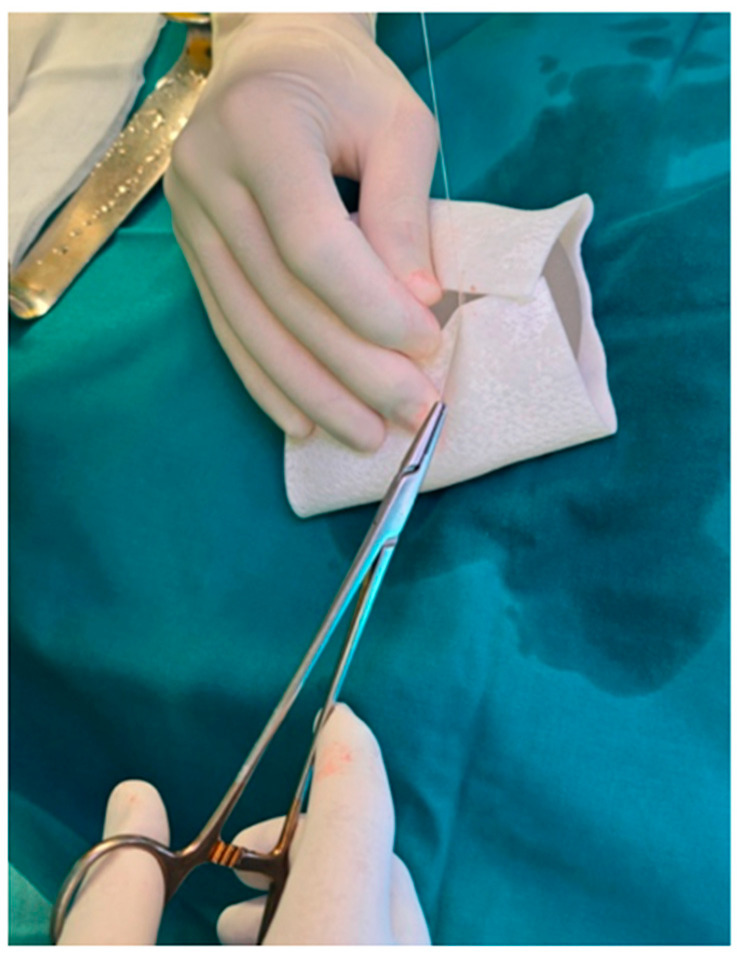
The breast implant was selected and wrapped in a collagen matrix before definitive implantation.

The selected implant was manually wrapped in the biocade^®^ collagen matrix using an envelope technique (Figure 3). The matrix was trimmed to size and rehydrated in room temperature sterile saline for a minimum of 5 min, per the manufacturer’s instructions. After rehydration, the ADM was folded around the implant and secured to itself using multiple interrupted absorbable sutures (4-0), ensuring full coverage and creating a stable construct. If not used immediately, the device can remain in sterile solution for up to 4 h if needed before surgical placement.

**Figure 4:**
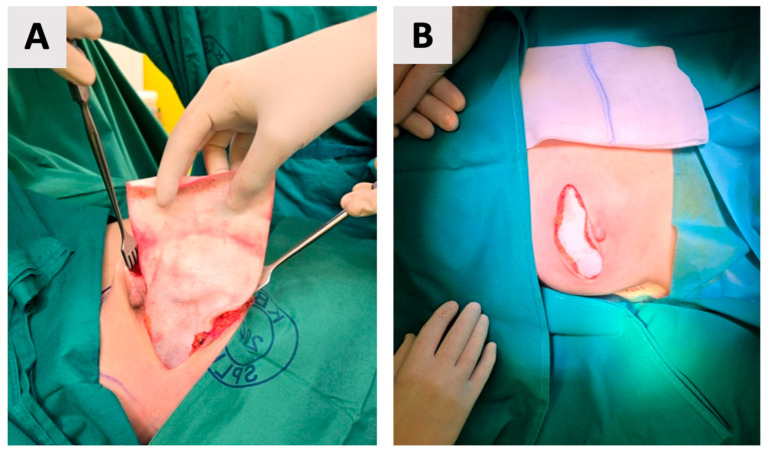
Placement of the wrapped implant into the prepared pocket. (**A**) Intraoperative view of the implant being positioned. (**B**) Final appearance before wound closure, with the skin ends approximated over the implant, ready for suturing.

The breast pocket was not irrigated with antiseptic or antibiotic solutions prior to implant placement. The implant was not anchored to the pectoralis major muscle, as the muscle was not elevated in this prepectoral approach. Instead, the ADM-implant construct was stabilized within a carefully dissected subcutaneous pocket that closely matched the implant dimensions. The ADM (biocade^®^) was secured to itself using multiple sutures, creating a firm and stable unit. Adequate adaptation of the pocket and mastectomy flap helped prevent implant displacement and minimized dead space (Figure 4).

**Figure 5:**
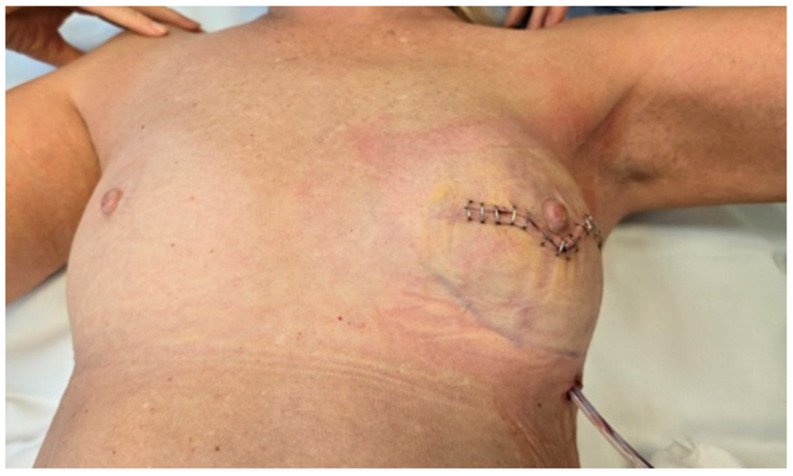
Final wound closure with suturing that minimize scarring. A drainage tube is visible, placed to manage postoperative fluid accumulation and reduce the risk of complications.

Following implant placement and ADM (biocade^®^) fixation, the surgical site was closed using standard suturing techniques for deeper layers, while skin staplers were used for epidermal approximation (Figure 5). This approach was selected for surgical efficiency and reliable skin edge alignment. A closed-suction drain was placed in the prepectoral pocket, positioned lateral to the ADM-wrapped implant. The drain exited through a separate incision to reduce the risk of contamination.

Postoperative care instructions were provided, and the patient received standard inpatient care, including gastroprotective therapy, low molecular-weight heparin for thromboprophylaxis, and analgesics as needed. Antibiotic therapy was not administered. Follow-up appointments were scheduled to monitor the recovery process and assess for any potential complications.

#### 2.2.4. Postoperative Course and Healing

**Figure 6:**
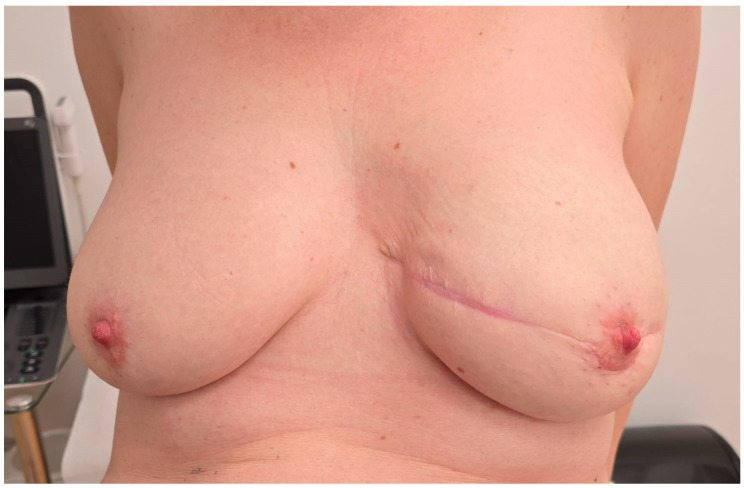
Clinical photograph taken at the 2-month follow-up visit. The surgical site shows proper wound healing, with no signs of infection, dehiscence, or delayed healing. The scar appears thin, well-aligned, and is maturing as expected. The overall aesthetic outcome of the reconstruction was satisfactory by both the patient and the surgical team.

Outcomes were assessed through direct clinical examination by the surgical team and based on the patient’s subjective satisfaction. No standardized aesthetic scales or validated questionnaires were used, consistent with the observational nature of this case report. Following surgery, the patient underwent an initial postoperative evaluation on the day of the procedure to address any acute concerns. The procedure was well tolerated, with no immediate complications observed. Postoperative care emphasized wound healing and monitoring for signs of infection or implant-related complications. The drain was monitored daily and removed once output was less than 30 mL for two consecutive days. No seroma or wound-related complications were observed following removal. During the first week, the patient was evaluated daily while hospitalized, followed by weekly outpatient follow-ups over the next two months. These visits confirmed satisfactory healing and progressive improvement in breast contour and symmetry. For two months follow-up (Figure 6), both the patient and the surgical team expressed satisfaction with the aesthetic outcome of the reconstruction. No visible scarring complications were reported during follow-up. No clinical signs of implant-related adverse events, such as seroma formation, capsular contracture, or delayed wound healing, were observed.

## 3. Discussion

The use of collagen matrices in breast reconstruction has shown to be a promising approach to improve aesthetic and functional outcomes by reducing complications such as capsular contracture and improving tissue integration [5]. This case represents the first reported clinical use of biocade^®^, a novel porcine-derived, non-crosslinked ADM, in the context of breast reconstruction. Its favorable handling characteristics and structural properties supported uneventful healing and a satisfactory aesthetic outcome. The integration behavior observed in this case is consistent with findings from preclinical studies, such as the in vivo work by Ren et al. [9], which demonstrated reduced inflammatory response and early vascularization in non-crosslinked collagen membranes. The absence of preservatives and cross-linking agents in biocade^®^ may thus contribute to improved cytocompatibility and a better immune response, which are critical for optimal integration in soft tissue reconstructive procedures [12,13].

Breast reconstruction after mastectomy is a critical aspect of treatment for many patients, offering both physical and psychological benefits. The primary methods of reconstruction include autologous tissue flaps and implants, with implant-based techniques being the most prevalent in the Western world [14,15]. Reconstruction is not only essential for post-mastectomy patients but also for those with genetic predispositions, such as breast cancer gene (BRCA) mutations, and in cases of breast reduction or gender-affirming surgeries [14,16]. The introduction of silicone implants in the 1970s marked a significant advancement, although complications such as capsular contracture remain a concern [17]. Autogenous tissue reconstruction from multiple areas of the body, such as the abdomen or back, offers an effective and aesthetically pleasing alternative for patients. However, this method can be more invasive and may not be suitable for all patients due to factors like body habitus or previous surgeries [18]. In contrast, acellular dermal matrices (ADMs), particularly those derived from porcine collagen, have emerged as a valuable alternative, significantly improving breast reconstruction outcomes by reducing capsular contracture rates and improving tissue integration [5,19]. The use of ADMs has been shown to reduce capsular contracture recurrence rates, and the prepectoral implant technique with ADM has better long-term outcomes than subpectoral techniques [19]. Numerous ADMs have been used successfully in immediate prepectoral breast reconstruction, including Braxon^®^, as reported by Santanelli di Pompeo et al., in a prospective cohort study [20]. While Braxon^®^ is a porcine-derived, preshaped ADM commonly used for full implant coverage, biocade^®^ is a flat, non-preshaped, non-fenestrated porcine collagen matrix requiring manual adaptation during surgery. Unlike many other ADMs, biocade^®^ is free from chemical cross-linking and preservatives, potentially contributing to reduced immunogenicity and favorable integration, as supported by preclinical findings. These distinctions may influence handling properties, resorption rates, and tissue response, warranting further comparative clinical evaluation in larger cohorts.

Breast reconstruction is not only a physical restoration but also a psychological journey for patients. Studies have shown that patient expectations often revolve around the appearance and outcome of the breasts, the physical impact of reconstruction, the process of care and recovery, and the psychosocial impact of the procedure. In the context of using collagen matrices, patient satisfaction can be enhanced by achieving better aesthetic outcomes and reducing complications such as capsular contracture. The immediate results and improved aesthetics offered by direct-to-implant reconstruction with ADMs align well with patient expectations for a more natural appearance and faster recovery [21]. The surgical approach to breast reconstruction can significantly impact patient outcomes and satisfaction [22,23]. Traditionally, breast reconstruction has been performed using a two-step method involving tissue expanders. This approach requires multiple surgeries: the first to place the expander, followed by gradual inflation to stretch the skin, and finally, a second surgery to replace the expander with a permanent implant. Although effective, this method can lead to discomfort, prolonged recovery, and unnatural positioning of the breast mound during the expansion phase [24]. In contrast, direct-to-implant reconstruction, also known as one-stage, involves placing a permanent implant immediately after mastectomy, often using an ADM to support the implant and improve tissue integration. This approach offers several advantages, including immediate results, improved aesthetics, and reduced pain, especially when full ADM coverage is used [25]. The use of ADMs in one-stage reconstruction helps to minimize complications such as capsular contracture and promotes better implant positioning and stability, which was described in presented case. However, when deciding the surgical approach, the team should keep in mind that both methods carry risks of complications, such as seromas, infections, and capsular contracture, with increased risks when radiotherapy follows surgery [26,27]. Despite these challenges, the one-stage approach with ADM has become increasingly popular due to its potential for improved outcomes and patient satisfaction, particularly in carefully selected patients [28,29].

Compared to other widely used ADMs in breast reconstruction, such as Braxon^®^ (porcine-derived, preshaped, non-crosslinked ADM), SurgiMend^®^ (fetal bovine-derived, non-crosslinked ADM), and AlloDerm^®^ (human-derived, decellularized and rehydrated ADM), biocade^®^ offers unique characteristics. It is a flat, porcine-derived, non-crosslinked ADM that is preserved by freeze-drying, requiring rehydration before use. Unlike Braxon^®^, it is not preshaped and must be manually adapted []. In contrast to AlloDerm^®^, which is human-derived and often rehydrated during manufacturing, biocade^®^ requires chairside rehydration [30]. SurgiMend^®^ and AlloDerm^®^ have been widely studied with long-term clinical data [25,31,32,33]. In that context, biocade^®^ remains under early clinical investigation, which emphasize the need for comparative trials to assess outcomes, integration behavior, and cost-effectiveness.

This case report provides for the first time the clinical use of a biocade^®^ in breast reconstruction; however, it is important to acknowledge its limitations. As with all case reports, the findings cannot be generalized. Therefore, larger-scale studies are warranted to assess its long-term safety, integration behavior, and patient-reported outcomes. In addition, future investigations should aim to compare its cost-effectiveness and clinical performance with other widely used ADMs, given the growing emphasis on value-based surgical care in reconstructive procedures.

## 4. Conclusions

In conclusion, this case report represents a significant contribution to the field of breast reconstruction and gives a novel perspective for both surgeons and scientists involved in tissue engineering. The successful outcomes demonstrate that the tissue responded favorably to the use of an ADM, specifically biocade^®^, during prepectoral direct-to-implant breast reconstruction, leading to optimal healing. This approach may be particularly beneficial in cases where nipple-sparing mastectomy is indicated, as demonstrated in this case report. However, it also highlights the need for further research to fully explore the potential of these biomaterial and address the challenges associated with their use, with the goal of improving the patients’ outcomes in breast reconstruction.

## Data Availability

The original contributions presented in this study are included in the article Further inquiries can be directed to the corresponding author.
